# Polyclonal antibodies towards abrin and ricin—design and potential application for mass spectrometry-based analysis of human biosamples

**DOI:** 10.1007/s00204-025-04132-x

**Published:** 2025-07-11

**Authors:** Aline C. Vollmer, Claudia Fecher-Trost, Martin Jung, Marnie Cole, Tilman F. Arnst, Veit Flockerzi, Lea Wagmann, Markus R. Meyer

**Affiliations:** 1 Experimental and Clinical Toxicology and Pharmacology, Center for Molecular Signaling (PZMS), PharmaScienceHub (PSH), Saarland University, 66421 Homburg, Germany; 2 Department of Medical Biochemistry and Molecular Biology, Saarland University, 66421 Homburg, Germany; 3 Experimental and Clinical Pharmacology and Toxicology, Center for Molecular Signaling (PZMS), Saarland University, Buildings 61.4 and 46, 66421 Homburg, Germany

**Keywords:** Abrin, Ricin, Polyclonal antibodies, Magnetic beads, Affinity column chromatography, Liquid chromatography-mass spectrometry

## Abstract

The analysis of highly toxic proteins such as abrin and ricin is challenging but comprehensive analytical methods are essential for their unambiguous identification after ingestion. This study pursued three primary aims at detecting abrin and ricin in human biosamples while ensuring that laboratory staff remain protected from direct exposure to these toxic proteins. First, two polyclonal antibodies (pAB) against specific peptides of abrin-A and ricin should be produced. Thereby, antibody epitope mapping was performed, which proved that both pAB recognize specifically their target peptide. Second, an affinity column chromatography-based assay was developed, and finally, the generated pAB should be tested using two different approaches (A and B) for their application in mass spectrometry (MS)-based bioanalytical workflows. Approach A used blood and urine samples submitted to the author’s laboratory after suspected ricin intake. Samples were prepared for nanoLC-MS analysis using affinity column chromatography, gel electrophoresis, and overnight trypsin digestion. Analysis resulted in the confirmation of ricin presence in both plasma and urine. Approach B included the enrichment of an abrin-A-peptide and ricin-peptide using affinity column chromatography directly followed by LC-Orbitrap MS with a detection limit of at least 5 ng/mL in plasma and validation according to international recommendations. Since approach B is much more time-efficient and can be applied throughout laboratories due to the less equipment required, this strategy deserves further focus, especially in a clinical setting.

**Supplementary Information:**

The online version contains supplementary material available at 10.1007/s00204-025-04132-x.

## Introduction

Abrin and ricin are among the most toxic and lethal proteins known regardless of the route of administration (Feldberg et al. 2021; Hansbauer et al. 2017). Current developments in ricin research were recently summarized (Bolt and Hengstler 2023). Due to the potential misuse as biological weapon, both proteins are mentioned on the U.S. Select Agents and Toxins list (CDC 2025). Abrin is contained in the seeds of *Abrus precatorius*, while ricin is found in the seeds of the castor bean plant *(Ricinus communis)* (Feldberg et al. 2021; Hansbauer et al. 2017). They are members of the type-II ribosome-inactivating protein family with lengths of aa 576 (ricin, UniProt accession number P02879) and aa 528 (abrin-A, UniProt accession number P11140), composed of two subunits (A- and B-chain), and connected by a disulfide bridge (Tam et al. 2017). Once the proteins have entered the cells through receptor-mediated endocytosis, the process of eukaryotic protein synthesis is disrupted leading to cell death. In humans, the lethal dose causing 50% mortality (LD_50_) after ingestion is estimated to be 22–25 µg/kg body weight for ricin and 0.1–1 µg/kg body weight for abrin. Clinical diagnosis is very challenging as initial symptoms appear 3–20 h after ingestion and are generally non-specific, including abdominal pain, vomiting, diarrhea, and nausea, leading to multi-organ failure and death (Chen et al. 2014a; Hansbauer et al. 2017; Janik et al. 2019; Worbs et al. 2011). There is currently no specific treatment, such as an antidote or vaccine, so treatment in hospital must be symptomatic and supportive (Rasetti-Escargueil and Avril 2023). Residual levels of e.g., ricin in rat sera have been reported as low as 10 ng/mL 12 h post-exposure, indicating the need of rapid detection of such toxins as concentrations decline rapidly (Feldberg et al. 2021; Ma et al. 2014).

Due to their ability to selectively recognize specific epitopes presented by an antigen, antibodies are versatile in research and clinical applications. It can be differentiated between monoclonal and polyclonal antibodies (mAB and pAB). The purity of mAB is usually higher but polyclonal sera contain a composition of pAB with unique specificity as they are produced by many B-cell clones, each generating antibodies against a specific epitope. pAB also provide greater resistance to changes in pH and salt concentrations and can be produced more rapidly (within several months) (Lipman et al. 2005). The decision whether to use mAB or pAB depends on factors such as (commercial) availability or the purpose of the application.

At present, detection methods for abrin or ricin are based on antibody-antigen interactions such as the enzyme-linked immunosorbent assay (ELISA) (Chen et al. 2014b; Feldberg et al. 2021; Garber et al. 2008). However, the complex and time-demanding workflow as well as the potential cross-reactivity between the enzyme-labeled secondary antibody and other sample proteins should be considered (Feldberg et al. 2021; Jiang et al. 2021). Liquid chromatography (LC)-high-resolution tandem mass spectrometry (HRMS/MS)-based analysis allows the unambiguous identification of proteins via unique peptides after enzymatic digestion. Nevertheless, this technique is susceptible to matrix interferences and is often not sensitive enough to detect substances in trace amounts without sufficient sample preparation (Li et al. 2024). Hoyt et al. described an assay based on ricin capture using magnetic beads followed by matrix-assisted laser desorption ionization (MALDI) time-of-flight (TOF) MS (Hoyt et al. 2021). Magnetic bead-based antibody column chromatography followed by LC-HRMS/MS analysis has also already been proven to sensitively enrich amatoxins down to 1 ng/mL from human urine (Vollmer et al. 2024). Therefore, this study aimed to (i) design pAB against specific peptides of abrin-A and ricin, (ii) develop an affinity column chromatography-based assay without the need for the toxic proteins as reference material, and (iii) evaluate the applicability of the generated pAB for MS-based analysis to detect abrin or ricin in human biosamples.

## Materials and methods

### Chemicals and reagents

The abrin-A-peptide and ricin-peptide sequences as given in Table 1 were synthesized by Pepmic (Suzhou, China). Peroxidase (POD)-coupled secondary anti‐rabbit antibodies from goat (A8275) were bought from Merck KGaA (Darmstadt, Germany). SulfoLink Coupling Resin, Dynabeads Protein A and G, PageRuler Plus Prestained Protein Ladder, DynaMag-2, Bolt 4–12% BT gradient gels, and Vivaspin 20 centrifugal concentrator (molecular weight cut-off, 100,000 Da) were bought from Fisher Scientific (Schwerte, Germany), Econo-Chromatography columns (1 × 10 cm) from Bio-Rad Laboratories (Feldkirchen, Germany), and sequencing grade modified trypsin from Promega (Walldorf, Germany). Acetonitrile (ACN), dimethyl sulfoxide (DMSO), sodium azide (NaN_3_), and sodium chloride (NaCl) were purchased from VWR International GmbH (Darmstadt, Germany), formic acid (LC–MS grade) and iodoacetamide (98%) from Fisher Scientific (Schwerte, Germany), 2-mercaptoethanol (≥ 99%), glycerol (≥ 99.5%), glycine (≥ 99%), sodium hydrogen carbonate (NaHCO_3_, ≥ 99%), tris-(hydroxymethyl)-aminomethane (TRIS) Pufferan^®^ (≥ 99.9%) from Carl Roth GmbH and Co. KG (Karlsruhe, Germany), and dithiothreitol (DTT) from AppliChem (Darmstadt, Germany). Polypropylene tubes were obtained from Greiner Bio-One GmbH (Frickenhausen, Germany), protein LoBind tubes and silanized vials from Eppendorf (Hamburg, Germany), and BD^™^ P100 blood collection tubes from BD Biosciences (Heidelberg, Germany). Water was purified with a Millipore filtration unit (18.2 Ω × cm water resistance) from Merck. Drug-free human pooled blank ethylenediaminetetraacetic acid (EDTA) plasma was obtained from a local blood bank.

**Table 1:** Liquid chromatography (LC)-Orbitrap characteristics of the abrin-A-peptide and ricin-peptide including the *m/z* (mass-to-charge) ratio of the most abundant positive charged fragment ions, retention time, and limit of identification (LOI)

Peptide sequence	Fragment ion, *m/z*	Retention time [min]	LOI [ng/mL]
Abrin-A-peptide			
CNPPNANQSPLLIRSIVEKSKI	[M + 3H]^3+^, 807.7809	3.69	5
	[M + 4H]^4+^, 606.0875		
Ricin-peptide			
CVYRCAPPPSSQFSLLIR	[M + 2H]^2+^, 1019.0240	3.68	5
	[M + 3H]^3+^, 679.6851		

### Generation of polyclonal antibodies

For generation of pAB against the abrin-A-peptide and ricin-peptide (pABAbrin and pABRicin), antisera were produced by repeated immunization of rabbits (AZ.2.4.1.1 and AZ.2.4.3.4) (Liewen et al. 2005; Philipp et al. 2000). The synthetic peptide corresponding to amino acids (C)V^291^YRCAPPPSSQFSLLIR^307^ of ricin (UniProt accession number P02879) with an additional amino-terminal cysteine and C^247^NPPNANQSPLLIRSIVEKSKI^268^ of abrin-A (UniProt accession number P11140) were conjugated to keyhole limpet hemocyanin and injected in rabbits with incomplete Freund´s Adjuvans. The sera were affinity purified using the synthetic peptides (see purification of polyclonal antibodies).

### Antibody epitope mapping

**Fig. 1:**
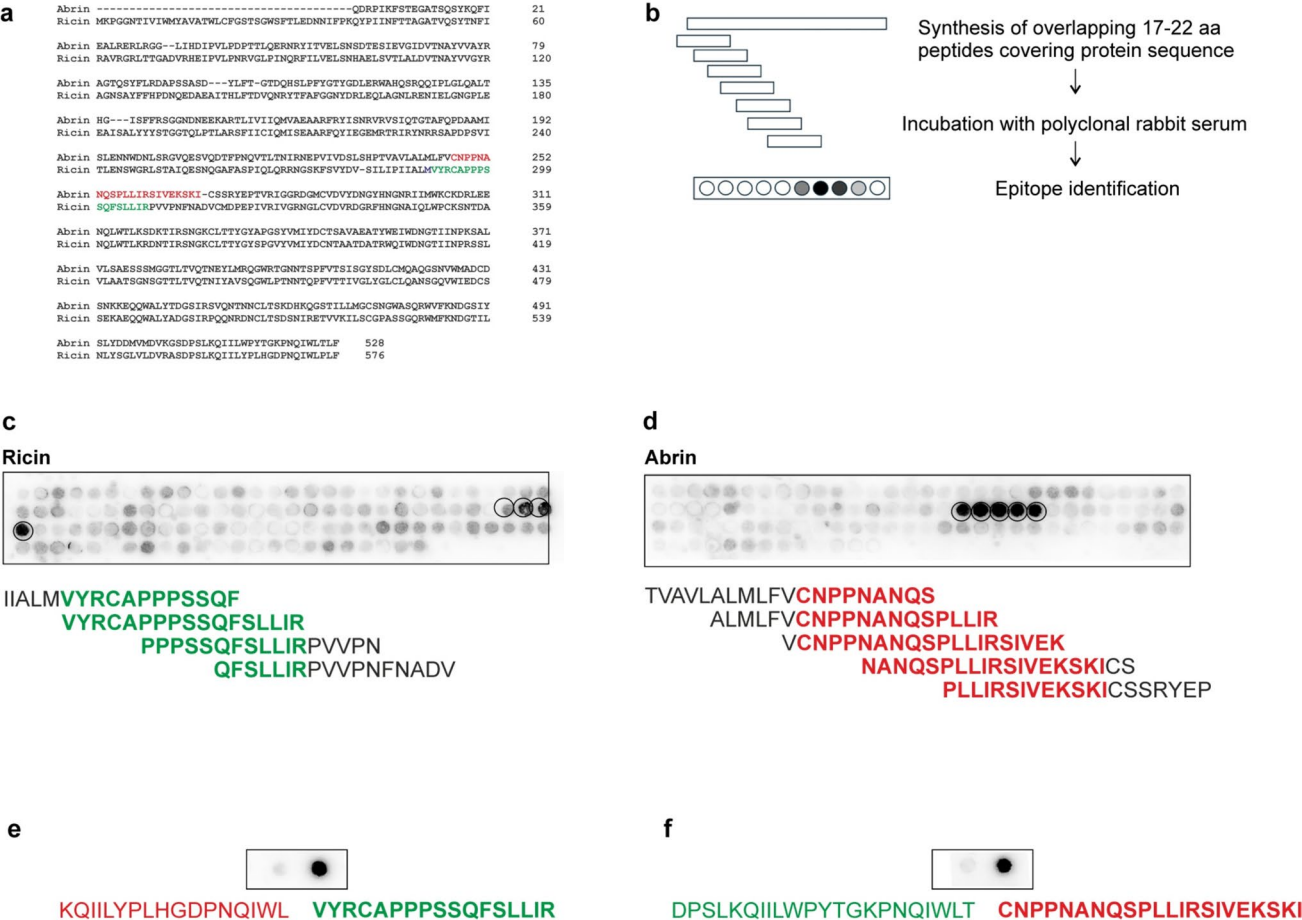
**a** Sequence alignment for abrin-A (P11140 · ABRA_ABRPR**)** and ricin (P02879 RICI_RICCO). Red: abrin-A-peptide sequence, green: ricin-peptide sequence. **b** General illustration of the antibody epitope mapping performed for the polyclonal antibodies (pAB) against the abrin-A-peptide and ricin-peptide. **c**–**f** Positive peptide spots were visualized after pABAbrin or pABRicin and secondary anti-rabbit antibody incubation by chemiluminescence. **c** Peptide blot incubated with pABRicin. Circled peptide spots indicate a strong reaction between the ricin-peptide and pABRicin. **d** Peptide array incubated with pABAbrin. Circled peptide spots indicate strong reaction between the abrin-A-peptide and pABAbrin. **e** For specificity testing, peptide blots were incubated with pABRicin. No reaction was detected between the abrin-A-peptide and pABRicin (left spot, negative control), strong reaction between the ricin-peptide with pABRicin (right spot, positive control). **f** Peptide blot incubated with pABAbrin. No reaction was detected between the ricin-peptide and pABAbrin (left spot, negative control), strong reaction between the abrin-A-peptide with pABAbrin (right spot, positive control)

Antibody epitope mapping was separately performed for pABAbrin and pABRicin using peptides immobilized on cellulose membranes based on a published procedure (Tirincsi et al. 2022). Peptides, covering the complete sequence of abrin-A or ricin with a length of 22 (abrin-A) or 17 (ricin) amino acid residues, were synthesized by a ResPep SL (Intavis) fully automated peptide synthesizer on a derivatized cellulose membrane via their C‐terminal ends. The amino acid frame was shifted by five amino acids from one spot to the following spot as shown in Fig. 1. In addition, peptides used for immunization with a length of 22 amino acids (aa 247-aa 268; abrin-A-peptide) or 17 amino acids (aa 291-aa 307; ricin-peptide) and a peptide (negative control, same length) from the respective proteins were synthesized. The membranes were activated using methanol (1 min, 22 °C) and washed with purified water followed by incubation with binding buffer (1% BSA in 150 mM NaCl, 50 mM TRIS–HCl, pH 7.5, 0.1% Triton X‐100) for 1 h under gentle shaking at 22 °C to minimize unspecific binding. The membranes were washed with binding buffer for 10 min each and, thereafter, incubated overnight at 4 °C with unpurified antibodies (1:2000 in binding buffer) against abrin-A and ricin, respectively. The incubation was followed by a washing step with binding buffer. For detection of the bound primary antibodies, POD‐coupled secondary anti‐rabbit antibodies (goat, 1:1000) were used, and the membrane was washed and subjected to luminescence imaging using ECLTM (GE Healthcare) and a Fusion SL imaging device (PEQLAB).

### Purification of polyclonal antibodies

Rabbit sera containing pABAbrin or pABRicin were affinity purified according to a published procedure with some modifications (Vollmer et al. 2024). For each pAB, an affinity column was prepared with the peptides that were also used for immunization. First, 15 mg of the abrin-A-peptide were dissolved in 3 mL coupling buffer (50 mM TRIS and 50 mM EDTA in purified water, pH 8.5) and 15 mg of the ricin-peptide were dissolved in 500 µL DMSO followed by the addition of 3 mL coupling buffer. Then, 2 mL of the SulfoLink Coupling resin were resuspended for 2 min (Vortex Genie 1 TouchMixer) and transferred to an Econo-Chromatography column followed by three washing steps using coupling buffer and the addition of the dissolved peptides. Two incubation steps were performed at 22 °C (30 min under gentle shaking and 30 min without shaking). After removal of each peptide solution and again two washing steps using 3 mL coupling buffer, 5 mL blocking solution (50 mM *L*-cysteine in coupling buffer) was added followed by two incubation steps at 22 °C as mentioned above. Afterwards, four washing steps using 4 mL coupling buffer and 4 mL 1 × TRIS-buffered saline (TBS, pH 7.4) were conducted. A volume of 5 mL rabbit serum each was diluted with 20 mL 1 × TBS in a polypropylene tube and applied onto the column overnight at 4 °C using a peristaltic pump (Pharmacia Biotech). The column resin was washed several times using 1 × TBS until the absorption A280 nm in the washing solution reached zero. Prior to the elution of the pAB, the ratio between the neutralization buffer (aqueous 0.1 M NaHCO_3_ containing 0.1 M NaCl, pH 8.5) and the elution buffer (100 mM aqueous glycine, pH 1.8) was tested to be pH 7.5. Finally, both pAB were eluted into 0.5 mL neutralization buffer using 2.5 mL aqueous glycine buffer. Absorption was measured from each eluate (1–5) using the NanoPhotometer N60. Eluates 1–3 were combined and concentrated in a Vivaspin 20 centrifugal concentrator via centrifugation (Sigma 3-16PK). After measuring the final concentration, purified pAB were stored in protein LoBind tubes at 4 °C. To keep and reuse the SulfoLink column, the resin was stored in 1 × TBS with NaN_3_ (0.05%) at 4 °C.

### Antibody affinity column preparation

Non-covalent binding of purified pABAbrin or pABRicin to Protein A and G magnetic beads as affinity ligands was performed according to a previously established procedure (Vollmer et al. 2024). Briefly, Dynabeads Protein A and G were resuspended by shaking for 1 min (VWR VV3, level 2.5) and 25 µL of each magnetic bead suspension were transferred to a protein LoBind tube and magnetized for 2 min using the DynaMag-2. After removal of the supernatant, two washing steps using 1 mL 1 × TBS were performed prior to the addition of 100 µL antibody working solution (see peptide and antibody solutions) and incubation for 1 h at 4 °C under gentle shaking (HLC Thermomixer KTMR-133). Finally, one further washing step using 1 mL 1 × TBS was performed.

### Gel electrophoresis

Based on a published procedure with some modifications, denaturing polyacrylamide gel electrophoresis was performed using Bolt 4–12% BT gradient gels (Fecher-Trost et al. 2013). The magnetic bead slurry with the immobilized antibody-antigen complex obtained after affinity column chromatography (see antibody affinity column preparation) was eluted with 30 µL denaturing sample buffer (120 mM TRIS HCl, pH 6.8, 8% SDS, 20% glycerol, and 0.72 M 2-mercaptoethanol) by incubation for 20 min at 60 °C (ThermoMixer HTMR-133) in a 1.5 mL protein LoBind tube. PageRuler Plus Prestained Protein Ladder was used as protein marker. Eluted proteins were separated for 10 min at 80 V and fixed with an aqueous mixture containing ethanol (40%, *v/v*) and acetic acid (10%, *v/v*) for 30 min at 22 °C. After three washing steps using purified water, proteins were visualized with colloidal Coomassie stain (methanol, 20%, *v/v*, phosphoric acid, 10%, *v/v*, ammonium sulfate, 10%, *w/v*, Coomassie G-250, 0.12%, *w/v*) for 30 min at 22 °C. Three washing steps were performed again as mentioned above. Stained areas were cut into three pieces and transferred separately to 1.5 mL protein LoBind tubes and alternatively washed twice with 300 µL solution A (50 mM NH_4_HCO_3_) and 300 µL solution B (50 mM NH_4_HCO_3_ and ACN 50%, *v/v*). After reduction for 30 min at 56 °C using 300 µL solution A^+^ (10 mM DTT) and alkylation for 30 min in the dark using 300 µL solution A^+^ (5 mM iodoacetamide), gel pieces were washed twice with 300 µL of solutions A and B alternating and placed in the vacuum centrifuge until they were dried completely. After overnight digestion at 37 °C using 15 µL trypsin (10 ng/µL), resulting peptides were extracted twice using 50 µL solution C each (ACN:purified water, 1:1, *v:v* containing formic acid, 2.5%, *v/v*) under sonication and completely evaporated in a vacuum centrifuge. Reconstitution was done in 21 µL aqueous formic acid (0.1%, *v/v*) followed by the analysis using nanoLC-trapped ion mobility spectrometry (tims) TOF MS (see nanoLC-timsTOF analysis of tryptic peptides).

### NanoLC-timsTOF analysis of tryptic peptides (approach A)

A nanoflow-based ultra-high performance LC (UHPLC) system (Bruker nanoElute, Bruker Daltonic, Bremen, Germany) connected to a timsTOF MS (timsTOF Pro 2, Bruker Daltonic) with a nano-electrospray ionization (ESI) source (nESI, CaptiveSpray) was used to analyze plasma and urine samples of suspected ricin intoxications. The instrument was calibrated according to the manufacturer’s recommendations. Three microliters of the extract were loaded on a PepMap^™^ Neo Trap Cartridge (300 µm × 5 mm, TF Scientific) and separated using a Thermo PapMap^™^ Neo column (150 mm × 75 µm, TF Scientific). Peptide elution was performed using eluent A (water with formic acid, 0.1%, *v/v*) and eluent B (ACN with formic acid, 0.1%, *v/v*). The gradient programmed, MS parameters, and the raw data evaluation using PEAKS are given in detail in Supplementary material S1 and S2.

### Peptide and antibody solutions

For preparation of the stock solutions, the abrin-A-peptide was dissolved in purified water and the ricin-peptide in DMSO (1 mg/mL, each). Dilutions of both peptides were prepared in purified water. For the establishment of the LC-Orbitrap-based approach, each peptide was separately and freshly spiked into blank plasma prior to affinity column chromatography (see affinity column chromatography). All solutions were handled in protein LoBind tubes and stored at − 20 °C. Both antibodies were affinity purified before application (see purification of polyclonal antibodies) and could be used for at least four months if stored at 4 °C. For antibody immobilization, a working solution containing 25 µL pABAbrin or pABRicin (25 µg/100 µL), 5 µL 20 × coupling buffer (10 mM Na_3_PO_4_, 150 mM NaCl, pH 7.2), 5 µL lysis and wash buffer (25 mM TRIS, 150 mM NaCl, 1 mM EDTA, 1% NP40, 5% glycerol, pH 7.4), and 65 µL purified water was used.

### Affinity column chromatography

The affinity binding of pABAbrin or pABRicin was then used to enrich the abrin-A-peptide and ricin-peptide from plasma, which corresponds to a previously reported procedure with minor modifications (Vollmer et al. 2024). A volume of 250 µL plasma fortified with the abrin-A-peptide or ricin-peptide was added to the pAB non-covalently immobilized onto the surface of the Protein A and G magnetic beads followed by incubation for 1 h at 22 °C under gentle shaking (800 rpm, CellMedia ThermoMixer TS pro). The plasma was discarded and four washing steps using 1 mL 1 × TBS were included. Elution of the bound peptides was performed by incubating with 150 µL aqueous glycine buffer for 5 min at 22 °C under gentle shaking, which was repeated three times. Afterwards, 400 µL of the final extract were pipetted into a fresh 1.5 mL protein LoBind tube of which 50 µL were transferred to a silanized vial followed by the injection of 10 µL onto the LC-Orbitrap system as described (see LC-Orbitrap analysis of peptides) and Supplementary material S3.

### LC-Orbitrap analysis of peptides (approach B)

Plasma samples spiked with the abrin-A-peptide or ricin-peptide were analyzed using a Thermo Fisher (TF) Vanquish Duo UHPLC system consisting of a degasser, a binary pump, and a dual split sampler HT (TF Scientific, Dreieich, Germany) coupled to a TF Orbitrap Exploris 120 system equipped with a heated ESI (HESI)-II source. The instrument was calibrated prior to analysis according to the manufacturer´s recommendations using external mass calibration. Gradient elution was performed on an Aeris Peptide XB-C_18_ column (150 mm × 2.1 mm, 1.7 µm particle size) using eluent A (water with formic acid, 0.1%, *v/v*) and eluent B (ACN:methanol, 1:1, *v:v*, plus water, 1%, *v/v*, and formic acid, 0.1%, *v/v*). The gradient programmed and MS parameters for the abrin-A-peptide and ricin-peptide analysis are described in Supplementary material S3.

### Method validation

The approach B was validated using 5 ng/mL spiked plasma samples (validation samples) in accordance with international guidelines and recommendations for qualitative analysis including selectivity, carry-over, matrix effects (ME), recoveries (RE), and different stability data (EMA 2022; Matuszewski et al. 2003). Limits of identification (LOI) were additionally determined (Peters et al. 2007; Wille et al. 2017). Validation experiments, acceptance criteria, as well as software used for data handling are described in detail in Supplementary material S4.

## Results and discussion

Antibody-based approaches such as ELISA, real-time cytotoxicity assay, ultrasound-assisted on-bead trypsin digestion, or immunoaffinity enrichment followed by MALDI MS for the identification of abrin or ricin particularly in food matrices were reported (Garber et al. 2008; Hansbauer et al. 2017; Jiang et al. 2021; Kull et al. 2010; Pauly et al. 2012; Simon et al. 2015). However, in a clinical setting, human biosamples such as plasma and urine are typically available for the determination of toxin ingestion (Hallbach et al. 2009). Data published by Wooten et al. and Johnson et al. focused on the detection of an abrin biomarker (*L*-methyl-*L*-tryptophan) in human and/or rat urine (Johnson et al. 2009; Wooten et al. 2014).

The workflow established by Drinkard et al. involved affinity capture based on the ability of the B-chain to bind glycoproteins. Following tryptic digestion, MALDI TOF MS was employed to uniquely identify peptides of abrin or ricin in trace amounts (Drinkard et al. 2024). As described, both proteins are not commercially available and require e.g., extraction from *Abrus precatorius* or *Ricinus communis* seeds (Drinkard et al. 2024; Feldberg et al. 2021; Hansbauer et al. 2017). Due to the high toxicity, experiments using the active abrin or ricin protein need to be conducted in a biosafety level-2 cabinet equipped with a High Efficient Particulate Air filter and all contaminated materials must be decontaminated with sodium hydroxide solution (2 M) (Jiang et al. 2021; Kreuzer et al. 2013). Since such equipment is often not provided in e.g., routine laboratories offering toxicological analysis, we aimed to detect abrin or ricin without the need to handle these toxic proteins in the laboratory.

**Fig. 2:**
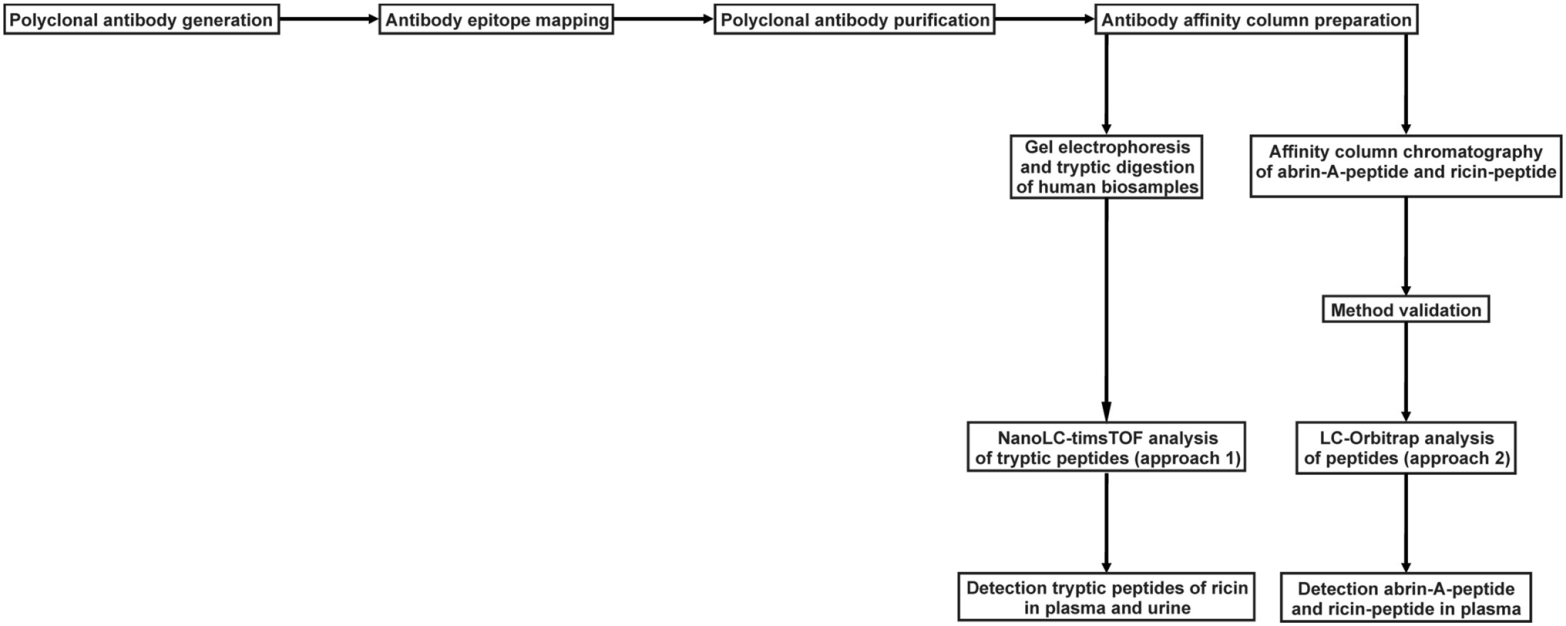
Workflow developed for the detection of abrin or ricin in human biosamples

Following the workflow illustrated in Fig. 2, rabbit pAB specifically against peptide structures of abrin-A and ricin were generated and investigated using two approaches including nanoLC-timsTOF analysis and patient biosamples (approach A) and LC-Orbitrap analysis (approach B) using an abrin-A and ricin related peptide for their potential implementation in the clinical setting.

### Peptide selection and characterization of polyclonal antibodies

Epitope mapping of pAB is commonly applied for the identification of antigenic moieties in sera of immunized animals. Thereby, immunodominant epitopes play an important role in toxin neutralization. Cohen et al. identified four immunodominant epitopes in the ricin protein, three in subunit A and one in subunit B, after epitope mapping using random phage display peptide libraries. Epitope 2 contained in the consensus sequence P^262^PSSQF^267^ has been reported to neutralize ricin (Cohen et al. 2014). Regarding abrin, four isoforms can be distinguished, abrin-A, abrin-B, abrin-C, and abrin-D with abrin-A exhibiting the highest toxicity with a reported LD_50_ of 2.11 µg/kg after intraperitoneal application in mice (Li et al. 2024). Alcalay et al. discovered 11 immunodominant epitopes on subunit A and 14 immunodominant epitopes on subunit B of the abrin protein. The abrin-A-peptide sequence selected for this study contains the immunodominant epitope sequence C^247^NPPN^251^ (Alcalay et al. 2020). Abrin and ricin are also related proteins sharing 46.2% identical amino acids (aa 268/576, Clustal Omega, Multiple Sequence Alignment). To produce pAB, two different peptides with lengths of 22 aa (abrin-A) and 17 aa (ricin) were selected within the centre of both proteins and containing the consensus sequences mentioned above. Both peptide sequences are shown in Table 1 and Fig. 1a.

Moreover, it is known that synthetic peptides must consist of at least six amino acids to be recognized as an antigenic epitope. Smaller peptides cause an immune response that is too weak e.g., no antibodies are produced towards the protein from which the peptides were derived (Harlow and Lane 1988). For further characterization and assessment of the specificity of pABAbrin and pABRicin, an epitope mapping was carried out using peptide blots as illustrated in Fig. 1b–f. As the amino acid sequence of the individual spots is known, it is possible to deduce the recognized epitopes of both antibodies. On the peptide blots, the complete abrin-A and ricin amino acid sequence as peptides with 22 and 17 amino acids each was synthesized on a membrane (dot), with neighbouring peptides overlapping by five amino acids (see Fig. 1b). The peptide blots were consecutively incubated with pABRicin or pABAbrin (rabbit sera, see Fig. 1c–f) and secondary anti-rabbit antibodies (see antibody epitope mapping). The ricin antibody recognizes four peptides seen as strongest signals covering aa 291–307 of the ricin protein (see Fig. 1c, green marked), whereas the abrin antibody recognizes peptide spots covering aa 247–268 of the abrin-A protein (see Fig. 1d, red marked). To test the specificity, pABRicin was also incubated with a peptide blot containing the ricin and a peptide from the C-terminus of ricin (left dot = negative control, see Fig. 1e). The corresponding negative control for the abrin antibody also proves the specificity of pABAbrin, as the peptide from the C-terminus of abrin was not recognized (see Fig. 1f, left dot = negative control). As these results showed that the sera contained specific antibodies, they were affinity purified and enriched with the immobilized antigenic peptide (see development of polyclonal abrin and ricin antibody affinity columns).

### Development of polyclonal abrin and ricin antibody affinity columns

Since this study aimed to develop a strategy to detect the presence of abrin or ricin in human biosamples without handling the toxic proteins, an antibody-based enrichment assay using affinity column chromatography together with LC–MS analysis was established. Therefore, pABAbrin and pABRicin were enriched and affinity purified with immobilized non-hazardous abrin-A-peptide or ricin-peptide from rabbit serum. Both affinity purified pAB were then incubated with protein A and G magnetic beads. To determine the final amount of pABAbrin or pABRicin used, different concentrations were tested (5 µg, 10 µg, 20 µg, and 25 µg pAB/100 µL) with respect to the detection of the target peptides using LC-Orbitrap analysis. This showed that 25 µg/100 µL were evaluated most suitable as illustrated by the extracted ion chromatograms given in Supplementary Fig. S1 and S2. Affinity columns prepared can at least be stored at 4 °C for at least six months. This time period covered method development and validation.

### Polyclonal antibodies—Proof-of-application

#### NanoLC-timsTOF analysis of human biosamples (approach A)

**Fig. 3:**
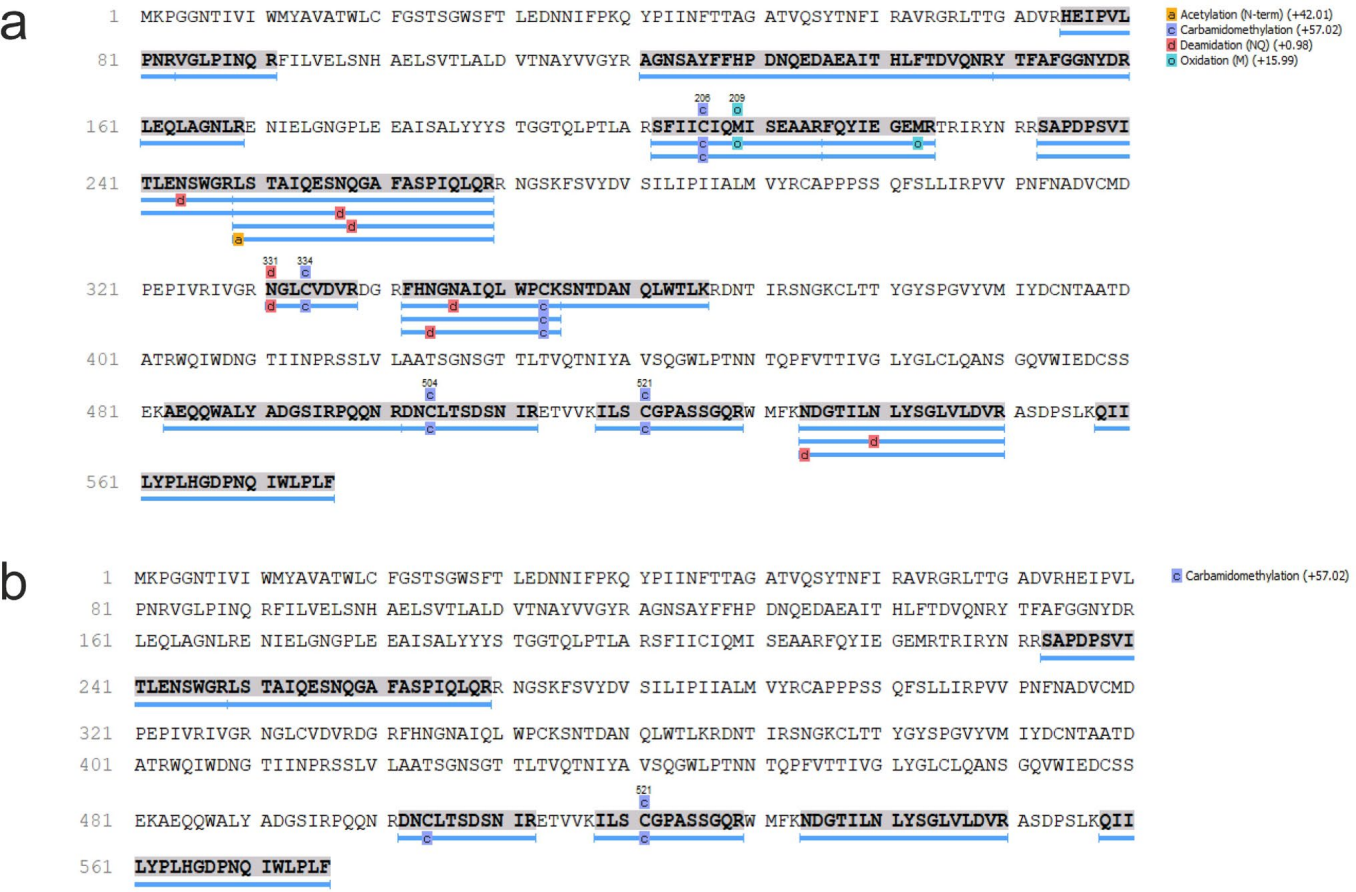
Detection of ricin in patient sample ID 1. Peptide sequences obtained for ricin identification in human plasma (**a**) and human urine (**b**) after data evaluation using PEAKS

Two human biosamples (sample ID 1 and 2) with suspected ricin intoxication submitted to the author´s laboratory for regular clinical toxicological analysis were used to investigate the suitability of pABRicin for protein capture in plasma and urine samples. The samples were first subjected to affinity column chromatography followed by enzymatic trypsin digestion and preparation for mass spectrometry analysis (see purification of polyclonal antibodies, antibody affinity column preparation, gel electrophoresis, nanoLC-timsTOF analysis of tryptic peptides, Supplementary material S1 and S2). After Swiss-Prot database search, ricin ingestion could be shown for sample ID 1 but not for sample ID 2 in both matrices by the identification of unique ricin-derived peptides. Peptide sequences of ricin identified are listed in Table 2 and illustrated in Fig. 3.

**Table 2:** Peptide sequences (*n* = 27) identified to confirm ricin detection after data evaluation using PEAKS

Peptide sequence
K.ILSCGPASSGQR.W
K.NDGTILNLYSGLVLDVR.A
R.SAPDPSVITLENSWGR.L
R.YTFAFGGNYDR.L
R.SFIICIQMISEAAR.F
K.SNTDANQLWTLK.R
R.SFIICIQMISEAAR.F
R.LSTAIQESNQGAFASPIQLQR.R
R.LSTAIQESNQGAFASPIQLQR.R
K.QIILYPLHGDPNQIWLPLF
K.AEQQWALYADGSIRPQQNR.D
R.DNCLTSDSNIR.E
R.HEIPVLPNR.V
R.SAPDPSVITLENSWGR.L
R.LSTAIQESNQGAFASPIQLQR.R
K.NDGTILNLYSGLVLDVR.A
R.AGNSAYFFHPDNQEDAEAITHLFTDVQNR.Y
R.FHNGNAIQLWPCK.S
R.FQYIEGEMR.T
R.LSTAIQESNQGAFASPIQLQR.R
R.FQYIEGEMR.T
R.FHN(+.98)GNAIQLWPCK.S
R.LEQLAGNLR.E
K.NDGTILNLYSGLVLDVR.A
R.NGLCVDVR.D
R.VGLPINQR.F
R.FHNGNAIQLWPCK.S

Cleavages after amino acid R (arginine) or K (lysine)

As shown in Fig. 1a, abrin-A and ricin share sequence homology. Therefore, we added the sequences of abrin-A and ricin to the human Swiss-Prot database used for data alignment to check for possible cross-reactivity between the pAB. Ricin but not abrin-A was identified for sample ID 1. Furthermore, both patient samples were also analyzed for ricinine according to published procedures (Bambauer et al. 2020; Maurer et al. 2023; Wissenbach et al. 2011).

Ricinine, which belongs to the group of piperidine alkaloids, is contained in small quantities in almost all parts of *Ricinus communis* and can also be used as biomarker for suspected *Ricinus communis* intake (Franke et al. 2019). It could be detected in both patient samples. However, its detection is not necessarily related to an ingestion of ricin, as ricinine is also present in ricin free *Ricinus communis* preparations as castor oil (Pittman et al. 2013). Thus, methods of detecting ricin in particular are necessary. However, approach A is time-consuming and takes three days, in particular due to the overnight enzymatic trypsin digestion. Ma et al. analyzed serum samples using magnetic beads coupled with anti-ricin mAB and tryptic digestion followed by LC-quadrupole TOF MS analysis. The entire procedure also took more than six hours, which is particularly important to consider in time-sensitive cases (Ma et al. 2014). To provide further evidence of the applicability of the generated pAB, we have developed an approach B with the aim to be more time-saving and to be adopted by other laboratories using a benchtop mass spectrometer.

#### LC-Orbitrap analysis of peptides (approach B)

After enrichment of the abrin-A-peptide and the ricin-peptide from plasma, reversed-phase chromatography using an Aeris Peptide XB-C_18_ column was evaluated for peptide detection (Hansbauer et al. 2017; Saadi et al. 2021). Separation was performed within 6 min total runtime followed by identification based on MS^2^ spectra and retention times. LC-Orbitrap characteristics including the LOI are summarized in Table 1. Full scan with data-dependent MS^2^ and product ion scan mode were evaluated. Product ion scan showed best sensitivity. Reconstructed ion chromatograms of the *m/z* of the abrin-A-peptide and ricin-peptide are depicted in Supplementary Fig. S1 and S2. An internal standard (IS) was not used in this approach to avoid reducing the capacity of target peptide binding, as the affinity column chromatography also would have to be applied for the IS.

#### Method validation (approach B)

Approach B was then validated for each peptide in plasma, as ricin and abrin enter the blood rapidly and are then distributed to other tissues such as the lungs, liver, or spleen and about 20% are excreted in the feces (Griffiths 2011). Blood is therefore best suited for the early diagnosis of suspected ricin or abrin ingestion (Cook et al. 2006). Selectivity was shown to be given for both peptides as no interferences were present at the respective retention time. Carry-over was not observed after injection of plasma samples spiked with a certain amount of the target peptide each (abrin-A-peptide, 30 ng/mL and ricin-peptide, 50 ng/mL). The presence of at least six scan points obtained after the product ion scan analysis was used to determine the LOI, which was evaluated down to 1 ng/mL. However, due to missing scan points at this concentration level, both target peptides were reproducibly identified in plasma until at least 5 ng/mL, indicated by coefficients of variations (CV) of 6.7% (abrin-A-peptide, *n* = 3) and 8.2% (ricin-peptide, *n* = 3). The achieved LOI is also in line with published data. For instance, Feldberg et al. developed an assay based on lectin affinity capture, which also enables ricin identification down to 5 ng/mL in human serum (Feldberg et al. 2021). Abrin isoforms as well as the agglutinin could be detected above 3 ng/mL using the procedure by Hansbauer et al. (Hansbauer et al. 2017). ELISA-based assays yielded detection limits equal or below 1 ng/mL (Simon et al. 2015; Tam et al. 2017).

ME for the abrin-A-peptide was calculated to be 103%. Although the ME is within an acceptable range of ± 25%, reproducibility needs to be discussed as the CV was calculated to be 31%. Ionization techniques such as HESI are susceptible to ME due to several mechanisms that occur in the ion source e.g., charge droplet formation, solvent evaporation, or droplet fission. Different approaches might help to minimize ME including the optimization of sample clean-up as well as the adjustment of MS and chromatographic conditions. Analysis using negative HESI mode should be preferred as less components giving response during ionization (Cortese et al. 2020). However, this was not applicable for the peptides as they were better detectable using positive ionization. Elution conditions e.g., the composition of the mobile phases or the pH value, as well as the use of stationary phases should also be carefully adjusted to the detection of the target compounds (Cortese et al. 2020; Trufelli et al. 2011). For sample clean-up, antibody-based enrichment allows for specific capture of target proteins or peptides and thus, a reduction of potential ME (Ascoli and Aggeler 2018).

The use of magnetic particles as solid support for antibody attachment proved to be suitable for immunoenrichment (Vollmer et al. 2024). Here, the Dynabeads Proteins A and G were used due to their high coupling efficiency and the fact that MS can be used as downstream application (Whiteaker et al. 2007). However, it should be considered that the extent of ME and its reproducibility is always compound-dependent, variable, and requires evaluation for each investigated analyte because in case of the ricin-peptide, ME was determined to be 101% with a reproducible CV of 10% (Cortese et al. 2020). RE for the abrin-A-peptide was calculated to be 24% (CV, 23%) and for the ricin-peptide to be 11% (CV, 14%). Although the RE are relatively low, reproducibility was given with both CVs ≤ 25%. Stock solution stability was given for both peptides over three weeks stored at − 20 °C as CVs were ≤ 15%.

Results of the stability experiments concerning processed samples, short-term, benchtop, and freeze and thaw stability performed in plasma after 24 h are summarized in Table 3, results obtained for the long-term stability are given in Table S1. In protein LoBind tubes, both peptides showed degradation under the tested storage conditions, as the criteria proposed by the ICH M10 guideline, whereby the mean concentration is not allowed to deviate more than ± 15% from the nominal concentration, were not fulfilled (EMA 2022). Therefore, peptide stability in protein LoBind tubes (short-term, benchtop, freeze and thaw) was re-evaluated over a shorter period (7 h), with the result that degradation was still an issue for both peptides. Results obtained after 7 h storage of the spiked plasma samples are given in Table S2. Blood plasma contains a variety of different proteases e.g., serin-, cysteine-, or aminopeptidases, which are responsible for the proteolytic degradation of proteins to peptides or amino acids by hydrolytic cleavage of peptide bonds, which may be a reason for the stability issues observed for the abrin-A-peptide and ricin-peptide (Bottger et al. 2017). Hence, short-term (24 h, 4 °C), benchtop (24 h, 22 °C), and freeze and thaw stability (24 h, − 20 °C) were tested again using tubes spray coated with K_2_EDTA anticoagulant and proteases inhibitors (BD^™^ P100 blood collection tubes). According to these results, presented in Table 3, plasma containing the abrin-A-peptide can be stored at − 20 °C for at least 24 h and the ricin-peptide can be stored at 4 °C or at − 20 °C for at least 24 h, which has to be taken into account especially during transport and storage of patient samples. To develop approach B more thoroughly without handling these toxic proteins, additional patient samples containing abrin or ricin are required, in particular to establish an optimized fast enzymatic digestion protocol.

**Table 3:** Stability data for the abrin-A-peptide and ricin-peptide. Spiked blank plasma samples stored in protein LoBind tubes under different conditions and processed samples stored in the autosampler (*n* = 3, 5 ng/mL each)

Peptide sequence	Validation sample, (CV, %)			
	Short-term (24 h, 4 °C)	Benchtop (24 h, 22 °C)	Freeze/thaw (24 h, − 20 °C)	Processed sample (24 h, 20 °C)
Abrin-A-peptide				
CNPPNANQSPLLIRSIVEKSKI	71 (15) *52 (12)*	27 (7.3) *20 (23)*	82 (4.1) *105 (19)*	101 (4.4)
Ricin-peptide				
CVYRCAPPPSSQFSLLIR	2.8 (27) *101 (3.7)*	1.8 (10) *83 (4.3)*	23 (2.2) *102 (2.6)*	97 (7.7)

Peak area deviations of measurement at timepoint t_0_ compared to t_1_, % and CVs, %. Stability results marked in italics are received using proteases inhibitor coated tubes. CV, coefficient of variation

#### Approach A and B—comparison, limitations, and outlook

The development of approach A and B demonstrated potential applications of pABAbrin and pABRicin, evidenced by their successful implementation in two different MS-based bioanalytical workflows as promising options for the analysis of suspected abrin or ricin intoxications.

However, approach A and B have advantages and disadvantages, which had to be addressed. Approach A indicated that pABRicin can selectively bind ricin. The time-consuming procedure, which includes the affinity column chromatography, the overnight trypsin digestion, and the comparably long MS analysis time of nearly 48 min needs to be considered, particularly in emergency situations. In contrast, approach B uses affinity column chromatography, short MS analysis time of 6 min, and the procedure and the analysis can be performed more widely throughout laboratories as the equipment required in approach A also limits its application. Different aspects need to be considered when handling these peptides. After evaluation of the stability data, proteases inhibitor coated tubes were recommended for sampling and storage until transport to the analytical laboratory, which are not available throughout the hospitals. Moreover, human samples contain the protein which means enzymatic digestion needs to be performed prior to MS analysis.

Despite these differences described for both approaches, pABAbrin and pABRicin demonstrated the ability to selectively bind peptides related to abrin-A or ricin. Established procedures often focused on the development of one targeted method for abrin or ricin detection. However, these approaches necessitate the use of sophisticated laboratory apparatus or involve time-consuming preparatory steps and MS analysis times of up to one hour (Hansbauer et al. 2017; Kalb et al. 2015; Ma et al. 2014). Therefore, two different MS-based approaches have been developed with approach B addressing these limiting factors. Furthermore, the implementation of the pAB in different MS-based workflows underscores their usefulness, versatility, and importance in the clinical context.

## Conclusions

In the present study, the successful design of pAB towards abrin and ricin was demonstrated. Both pAB proved to be useful for affinity column chromatography followed by MS-based analysis. Two approaches were investigated to detect the presence of abrin or ricin in human biosamples without exposure to the toxic proteins. The time-consuming approach A identified ricin in one of two suspected intoxications. Using approach B, target peptide identification was possible until at least 5 ng/mL. The procedure was validated in accordance with international guidelines and recommendations and revealed that peptide stability must be taken into account. Although enzymatic digestion is not yet included, this strategy allows the detection of peptides derived from highly toxic proteins within 80 min using a benchtop mass spectrometer, making this approach worth further investigating in the clinical setting, especially for emergency toxicology.

## Supplementary Information

Below is the link to the electronic supplementary material.

Supplementary file1 (PDF 390 KB)

## Data Availability

Data will be made available on request.
