# Supplementary material for: Polyclonal antibodies towards abrin and ricin—design and potential application for mass spectrometry-based analysis of human biosamples
Source: Arch Toxicol. 2025 Jul 11;99(11):4399–410. doi: 10.1007/s00204-025-04132-x (PMC12477077; doi:10.1007/s00204-025-04132-x)
Supplement: Supplementary file 1 — Supplementary file1 (PDF 390 KB) [file 204_2025_4132_MOESM1_ESM.pdf]

Supplementary Material

Polyclonal antibodies towards abrin and ricin – Design and potential application for mass spectrometry-based analysis of human biosamples

Aline C. Vollmer[a], Claudia Fecher-Trost[a], Martin Jung[b], Marnie Cole[c], Tilman F. Arnst[a], Veit Flockerzi[c], Lea Wagmann[a], and Markus R. Meyer[a]*

[a]Experimental and Clinical Toxicology and Pharmacology, Center for Molecular Signaling (PZMS), PharmaScienceHub (PSH), Saarland University, 66421 Homburg, Germany

[b]Department of Medical Biochemistry and Molecular Biology, Saarland University, 66421 Homburg, Germany

[c]Experimental and Clinical Pharmacology and Toxicology, Center for Molecular Signaling (PZMS), Saarland University, Buildings 61.4 and 46, 66421 Homburg, Germany

S1 NanoLC-timsTOF analysis of tryptic peptides (approach A)

The gradient was programmed as follows: 0-45 min from 2% B up to 35% B, 45-45.5 min up to 95% B, and 45.5-47.55 min hold 95% B. The flow rate was set to 0.4 µL/min and the column temperature to 50 °C. Main parameters were: polarity, positive; mass range from, 100 *m/z*; 1/K0 Start, 0.60 Vs/cm^2; rolling average, on; TIMS enable, on; scan mode, Parallel Accumulation Serial Fragmentation (PASEF); mass range to, 1700 *m/z*; 1/K0 End, 1.60 Vs/cm^2; rolling average No. 10. General parameters were: denoising mode, moderate; mass spectra peak detection, use maximum intensity; intensity threshold, absolute; absolute threshold, 10; intensity threshold, 5000.0. TIMS mode parameters were: intraclass correlation, off; imeX mode, custom; 1/K0 start, 0.60 Vs/cm^2; ramp time, 100.0 ms; advanced parameters, on; lock accumulation to mobility range, on; accumulation time, 2.0 ms; target, 2.0 Mio; resolution, custom; spectra rate, n/a Hz; lock duty cycle to 100%, on. Source parameters were: capillary, 1600 V; dry gas, 3.0 l/min; divert valve, waste1-6; nanoBooster, off; dry temperature, 180 °C. General tune parameters were: deflection 1 delta, 70.0 V; funnel 1 radio frequency (RF), 300.0 Vpp; ion energy, 5.0 eV; collision energy, 10.0 eV; transfer time, 60.0 µs; high sensitivity detection, off; funnel 2 RF, 200.0 Vpp; multipole RF, 500.0 Vpp; low mass, 200.00 *m/z*; collision RF, 1500.0 Vpp; pre pulse storage, 12.0 µs; stepping, off. Parameters for receiving PASEF data were: No. of PASEF MS/MS scans, 10; target intensity, 20000; active exclusion, on; reconsider precursor, if, on; intensity threshold, 2500; charge range maximum, 5; release after, 0.40 min; current intensity/previous intensity, 4.00. PASEF-collision-induced dissociation parameters: advanced collision energy settings, off; isolation mass start, 700.00 *m/z*; isolation width start, 2.00 *m/z*; 1/k0 Start, 0.60 V s/cm^2; energy start, 20.00 eV; isolation mass end, 800.00 *m/z*; isolation width end, 3.00 *m/z*; 1/k0 end, 1.60 V s/cm^2; energy end, 59.00 eV. PASEF-advanced parameters: MS repetitions, 1 x; summation width, 25 pts; mass width, 0.015 *m/z*; measuring time, 2.75 ms; cycle overlap, 4; maximum No. of peaks, 3; 1/K0 width, 0.015 V s/cm^2; switching time, 1.65 ms.

S2 Search parameters using PEAKS

Library search to identify ricin was carried out using the PEAKS Studio software (version 10.6 build 20201221) with the following parameters: search engine name, PEAKS; parent mass error tolerance, 15.0 ppm; fragment mass error tolerance, 0.1 Da; precursor mass search type, monoisotopic; enzyme, trypsin; maximum missed cleavage, 3; digest mode, specific; fixed modifications, carbamidomethylation, 57.02; variable modifications; oxidation (M), 15.99, acetylation (protein N-term), 42.01, and deamidation (NQ), 0.98; maximum variable post-translational modification per peptide, 5; database, human including the ricin and abrin sequence (taken from UniProt, P02879 · RICI_RICCO, P11140 · ABRA_ABRPR); taxon, all; searched entry, 20400; false discovery rate estimation, enabled; merge options, merged; precursor options; corrected; charge options, no correction; filter charge, 2-8; process, true; associate chimera, no.

S3 LC-Orbitrap analysis of peptides (approach B)

The gradient was programmed as follows: 0-1 min hold 1 % B, 1-4 min up to 99 % B, 4-4.5 min hold 99 % B, and 4.51-6 min down to 1 % B. The flow rate was set to 300 µL/min from 0-4.5 min followed by decreasing to 50 µL/min until 6 min. Heated electrospray ionization (HESI)-II source conditions were as follows: sheath gas, 70 arbitrary units; auxiliary gas, 10 AU; spray voltage, 4.00 kV (positive polarity); vaporizer temperature, 320°C; ion transfer capillary temperature, 320°C; and S-lens radio frequency level, 70.0. Settings for the product ion scan

mode were as follows: resolution, 30,000; scan range mode, define first mass; first mass, *m/z*, 200; normalized automatic gain control, 50 %; maximum injection time, 250 ms; Q1 resolution, *m/z*, 4. The injection volume was 10 µL.

S4 Data handling and method validation

Statistical analysis was performed using Microsoft Excel 2010 (Redmond, WA, USA). ACD/Chem Sketch freeware version 2015 was used for the calculation of the exact masses of the abrin-A-peptide and ricin-peptide sequences and TF Xcalibur Qual Browser version 4.1 for qualitative data evaluation. Qual Browser settings were as follows: detector type, MS; peak algorithm, genesis; plot type, mass range; *m/z* of each substance; mass tolerance, 5 ppm; mass precision, 4 decimals. Extracted ion chromatograms of the analytes were edited using CorelDraw X7 Version 17.0.0.491 (Munich, Germany). Graphical illustrations were created using BioRender (2023). The abrin-A-peptide and ricin-peptide detection using LC-Orbitrap analysis was validated according to international guidelines and recommendations covering different parameters [1-3]. Selectivity testing was performed using plasma samples from six different donors which were extracted, analyzed, and evaluated for interferences. An extracted plasma sample spiked with 30 ng/mL of the abrin-A-peptide and a plasma sample spiked with 50 ng/mL of the ricin-peptide was analyzed for carry-over followed by the injection of six extracted blank plasma samples each. Matrix effects (ME) and recoveries (RE) were determined according to Matuszewski et al. using three different sample sets (n = 6, concentration of the abrin-A-peptide or ricin-peptide, 5 ng/mL) [4]. The first sample set (neat standard) was prepared in aqueous glycine buffer (100 mM, pH 1.8). Blank plasma was spiked with the abrin-A-peptide or ricin-peptide before affinity column chromatography (sample set 3) or after affinity column chromatography (sample set 2). For the abrin-A-peptide and ricin-peptide, ME were calculated using the ratio of the peak area in the presence of matrix (sample set 2) and the peak area in absence of matrix (sample set 1). Division of mean peak areas of sample set 2 by those of sample set 1 were used for calculation of the ME. Coefficients of variation (CV) for both peptides should not be greater than 25 %. Division of mean peak areas of sample set 3 by those of sample set 2 was used for calculation of the RE. Stock solution stability of the abrin-A-peptide and ricin-peptide was tested over three weeks in purified water (n = 3, concentration of the abrin-A-peptide and ricin-peptide, 100 ng/mL). CVs not greater than ± 15 % were defined to be acceptable. Processed sample stability was investigated after 5 h, 10 h, 15 h, and 24 h (extracts stored at 20 °C; n = 3, concentration of the abrin-A-peptide and ricin-peptide, 5 ng/mL) next to short-term stability (spiked plasma samples stored 24 h at 4 °C), benchtop stability (spiked plasma samples stored 24 h at 22 °C), and long-term stability (spiked plasma samples stored three weeks at -20 °C). One freeze and thaw cycle was conducted for the freeze and thaw stability (spiked plasma samples stored for 24 h at -20 °C). Plasma samples spiked with the abrin-A-peptide or ricin-peptide were extracted and analyzed immediately after preparation (t_0) and after the appropriate storage condition (t_1). Peak area deviations as well as CVs not greater than ± 15 % were defined to be acceptable. Stability experiments were performed using protein LoBind tubes except for the processed sample stability where extracts were stored in silanized vials. Additionally, short-term, benchtop, and freeze and thaw stability over a period 24 h was investigated also using proteases inhibitor coated tubes.

References

1. Wille, S.M.R., et al., Update of Standard Practices for New Method Validation in Forensic Toxicology. Curr Pharm Des, 2017. **23**(36): p. 5442-5454.
2. Peters, F.T., O.H. Drummer, and F. Musshoff, Validation of new methods. Forensic Sci Int, 2007. **165**(2-3): p. 216-24.
3. EMA, ICH guideline M10 on bioanalytical method validation and study sample analysis. 2022, European Medicines Agency: Amsterdam, The Netherlands.
4. Matuszewski, B.K., M.L. Constanzer, and C.M. Chavez-Eng, Strategies for the assessment of matrix effect in quantitative bioanalytical methods based on HPLC-MS/MS. Anal Chem, 2003. **75**(13): p. 3019-30.

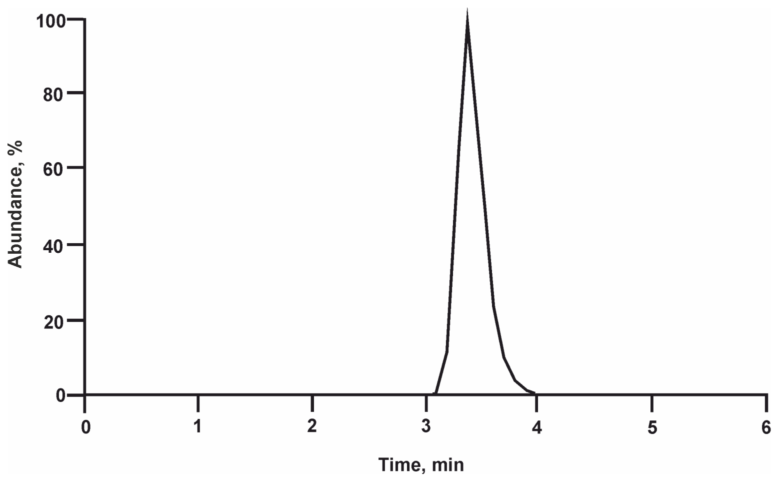

Fig. S1 Reconstructed ion chromatogram (*m/z*) of the ricin-peptide spiked in blank human plasma (5 ng/mL), extracted using 25 µg/100 µL pABRicin followed by liquid chromatography (LC)-Orbitrap analysis. All peaks at 100% abundance.

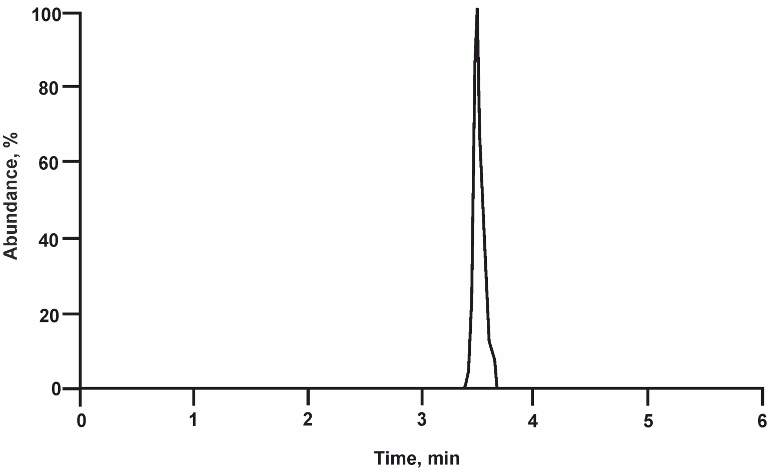

Fig. S2 Reconstructed ion chromatogram (*m/z*) of the abrin-A-peptide spiked in blank human plasma (5 ng/ml), extracted using 25 µg/100 µL pABAbrin followed by liquid chromatography (LC)-Orbitrap analysis. All peaks at 100% abundance.

Table S1 Long-term stability data for the abrin-A-peptide and ricin-peptide. Spiked blank plasma samples stored in protein LoBind tubes (n = 3, 5 ng/mL). Peak area deviations of measurement at timepoint t_0 compared to t_1, % and CVs, %. CV, coefficient of variation.

**Peptide sequence**	**Validation sample, (CV, %)**
	**Long-term (three weeks, -20 °C)**
**Abrin-A-peptide**	
CNPPNANQSPLLIRSIVEKSKI	69 (12)
**Ricin-peptide**	
CVYRCAPPPSSQFSLLIR	68 (17)

Table S2 Stability data for the abrin-A-peptide and ricin-peptide. Spiked blank plasma samples stored in protein LoBind tubes under different conditions for 7 h (*n* = 3, 5 ng/mL, each). Peak area deviations of measurement at timepoint t_0 compared to t_1, % and CVs, %. CV, coefficient of variation.

**Peptide sequence**	**Validation sample, (CV, %)**		
	**Short-term (7 h, 4 °C)**	**Benchtop (7 h, 22 °C)**	**Freeze/thaw (7 h, -20 °C)**
**Abrin-A-peptide**			
CNPPNANQSPLLIRSIVEKSKI	66 (15)	43 (33)	104 (5.9)
**Ricin-peptide**			
CVYRCAPPPSSQFSLLIR	3.4 (14)	3.8 (92)	51 (14)
